# Analgesic and Anesthetic Efficacy of Rocuronium/Sugammadex in Otorhinolaryngologic Surgery: A Propensity Score-Matched Analysis

**DOI:** 10.3390/ph15070894

**Published:** 2022-07-19

**Authors:** En-Bo Wu, Chao-Ting Hung, Sheng-Dean Luo, Shao-Chun Wu, Tsung-Yang Lee, Jo-Chi Chin, Peng-Neng Tsai, Johnson Chia-Shen Yang

**Affiliations:** 1 Department of Anesthesiology, Kaohsiung Chang Gung Memorial Hospital, Chang Gung University College of Medicine, No. 123, Ta-Pei Rd., Niao-Song Dist., Kaohsiung City 833, Taiwan; enbofive@gmail.com (E.-B.W.); timtim0070@gmail.com (C.-T.H.); shaochunwu@gmail.com (S.-C.W.); a0575@cgmh.org.tw (P.-N.T.); 2 Department of Otolaryngology, Kaohsiung Chang Gung Memorial Hospital, Chang Gung University College of Medicine, Kaohsiung City 833, Taiwan; rsd0323@cgmh.org.tw; 3 Graduate Institute of Clinical Medical Sciences, College of Medicine, Chang Gung University, Taoyuan 333, Taiwan; 4 Department of Anesthesiology, Jen-Ai Hospital, Taichung 412, Taiwan; jason721025@gmail.com; 5 Department of Anesthesiology, Park One International Hospital, No. 100, Bo’ai 2nd Rd., Zuoying Dist., Kaohsiung City 813, Taiwan; jochi731@gmail.com; 6 Division of Plastic and Reconstructive Surgery, Department of Surgery, Kaohsiung Chang Gung Memorial Hospital, College of Medicine, Chang Gung University, No. 123, Ta-Pei Rd., Niao-Song Dist., Kaohsiung City 833, Taiwan

**Keywords:** opioid-sparing anesthesia, otorhinolaryngologic surgery, propensity score analysis, sugammadex, volatile-sparing anesthesia

## Abstract

The use of rocuronium/sugammadex in otorhinolaryngologic surgery improves intubation conditions and surgical rating scales. This study primarily aimed to evaluate the effect of the combination of rocuronium and sugammadex on intraoperative anesthetic consumption. The secondary outcomes were the intraoperative and postoperative morphine milligram equivalent (MME) consumption, duration of intraoperative hypertension, extubation time, incidence of delayed extubation and postoperative nausea and vomiting, pain score, and length of stay. A total of 2848 patients underwent otorhinolaryngologic surgery at a tertiary medical center in southern Taiwan. After applying the exclusion criteria, 2648 of these cases were included, with 167 and 2481 in the rocuronium/sugammadex and cisatracurium/neostigmine groups, respectively. To reduce potential bias, 119 patients in each group were matched by propensity scores for sex, age, body weight, and type of surgery. We found that the rocuronium/sugammadex group was associated with significant preservation of the intraoperative sevoflurane and MME consumption, with reductions of 14.2% (*p* = 0.009) and 11.8% (*p* = 0.035), respectively. The use of the combination of rocuronium and sugammadex also significantly increased the dose of intraoperative labetalol (*p* = 0.002), although there was no significant difference in intraoperative hypertensive events between both groups. In conclusion, our results may encourage the use of the combination of rocuronium and sugammadex as part of volatile-sparing and opioid-sparing anesthesia in otorhinolaryngologic surgery.

## 1. Introduction

Neuromuscular blockade agents (NMBAs) are widely administered in otorhinolaryngologic surgery to facilitate endotracheal intubation and optimize surgical conditions. Similarly, surgical procedures that require delicate techniques within a limited surgical field require profound neuromuscular block with a higher dose of NMBA [1]. However, this raises concerns about residual neuromuscular blockade, which is associated with increased pulmonary complications, including impaired pharyngeal function [2], atelectasis [3], pneumonia [4], respiratory distress, and subsequent reintubation [5]. Consequently, clinicians must ensure the enhanced recovery of patients from NMBA to reduce the risk of respiratory complications. However, neostigmine, a conventional reversal, may have a limited effect in antagonizing deep neuromuscular block [6]. As an alternative to neostigmine, sugammadex can promptly antagonize neuromuscular blockade induced by steroidal NMBAs. In contrast to neostigmine, sugammadex binds to rocuronium or vecuronium in an equivalent ratio, antagonizes its neuromuscular blocking effect, and undergoes renal elimination by an NMBA–sugammadex complex, even in the presence of a profound neuromuscular block [7]. Reversal with sugammadex has been shown to lower the incidence of residual paralysis [8] and major pulmonary complications [9].

The use of sugammadex with deep neuromuscular block in elective laryngeal microsurgery (LMS) has been shown to improve intubation conditions and the surgical rating scale [10]. Sugammadex, used as a reversal agent, has also been shown to have significantly shorter extubation time and fewer tachycardia events in the post-anesthesia care unit (PACU) than pyridostigmine in elective LMS [11]. Moreover, other studies have shown that reversal with sugammadex results in less diaphragmatic failure [12] and less postoperative desaturation and hypoxemia [13]. However, the relationship between the combination of rocuronium and sugammadex and intraoperative anesthetic consumption in otorhinolaryngologic surgery has not been well established. Therefore, this study aimed to evaluate the effect of rocuronium on intraoperative sevoflurane consumption. We also investigated events of intraoperative hypertension, doses of antihypertensive agents, time to extubation, incidence of delayed extubation, assessment of postoperative pain, incidence of postoperative nausea and vomiting (PONV), and length of stay (LOS).

## 2. Results

**Figure 1:**
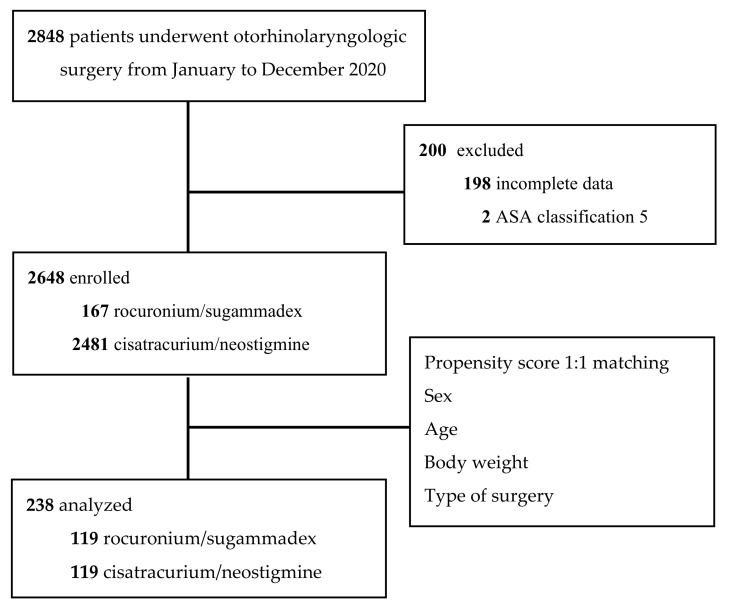
Flow diagram of the study participants. ASA, American Society of Anesthesiologists physical status classification.

From a total of 2848 patients who underwent otorhinolaryngologic surgery at our institution in 2020, 200 patients were excluded, and 2648 patients were enrolled. A 1:1 propensity score-matched analysis of sex, age, body weight, and type of surgery was performed for 238 patients, with 119 patients in the cisatracurium/neostigmine group and 119 patients in the rocuronium/sugammadex group (Figure 1).

The demographic characteristics of the study cohort in the different perioperative phases are summarized in Table 1 and Table 2. Sex, age, body weight, the American Society of Anesthesiologists (ASA) physical status classification, Apfel score, hypertension, diabetes mellitus, cerebrovascular accident, and surgical indication for otorhinolaryngologic surgery were not significantly different between the cisatracurium/neostigmine and rocuronium/sugammadex groups (Table 1). Intraoperative variables, which included baseline mean arterial pressure (MAP), percentage of MAP > 120% at 5-min intervals, and fluid, also showed no significant difference between the two groups. Moreover, there were no significant differences in the postoperative variables between the two groups, including the extubation time, percentage of delayed extubation, postoperative dose of parecoxib, postoperative morphine milligram equivalent (MME) consumption, visual analog scale (VAS) and PONV in the ward, blood loss, and LOS (Table 2).

The duration of anesthesia was significantly shorter in the cisatracurium/neostigmine group than in the rocuronium/sugammadex group (2.82 vs. 4.63 h, *p* < 0.001). Sevoflurane consumption (0.18 vs. 0.21 mL/h) and intraoperative MME consumption (0.067 vs. 0.076 mg/kg/h) were significantly less in the rocuronium/sugammadex group than the cisatracurium/neostigmine group (*p* = 0.009 and *p* = 0.035, respectively). In addition, the dose of intraoperative labetalol was significantly higher in the rocuronium/sugammadex group (*p* = 0.002), although there was no significant difference in intraoperative hypertensive events (*p* = 0.325) between both groups. Another antihypertensive agent, nicardipine, did not show a significant difference between the two groups (*p* = 0.245).

## 3. Discussion

In this retrospective single-center study comparing rocuronium/sugammadex and cisatracurium/neostigmine in adults undergoing otorhinolaryngologic surgery, we found that the use of rocuronium/sugammadex during anesthesia was associated with a significant reduction in the hourly consumption of sevoflurane (*p* = 0.009) and intraoperative MME (*p* = 0.035). No significant differences were found between the two groups in terms of intraoperative hypertensive events, dose of postoperative analgesics, VAS score, extubation time, incidence of delayed extubation and PONV, or LOS. There was a significant difference in the dose of intraoperative labetalol (*p* = 0.002).

Several studies have compared the outcomes of using different neuromuscular blocking drugs and their reversal agents in otorhinolaryngologic surgery [10,11,12,14]. Our study is the first to investigate the hourly consumption of sevoflurane. Our study showed that the combination of rocuronium and sugammadex significantly reduced the hourly consumption of sevoflurane. This may be because anesthesiologists are more willing to maintain a deep to intense neuromuscular block when sugammadex is used as the reversal agent and also reflects the preference of anesthesiologists to use the combination of rocuronium and sugammadex for longer procedures. Our results also showed that the duration of anesthesia was significantly longer in the rocuronium/sugammadex group than in the cisatracurium/neostigmine group (4.63 h vs. 2.82 h, *p* < 0.001).

Sevoflurane is a halogenated ether and volatile anesthetic that disrupts consciousness and cognition and is used to maintain the depth of anesthesia during surgery [15]. Besides its hypnotic effect, it has been shown to enhance the neuromuscular blocking effect of NMBAs [16,17,18]. Bevan et al. demonstrated that the total dose of NMBA was significantly reduced under qualitative neuromuscular monitoring in the presence of sevoflurane [19]. This may indirectly imply that sevoflurane consumption can be reduced in the presence of deeper neuromuscular blockade intraoperatively. Another study that was inconsistent with the current study showed that the administration of sugammadex as a reversal agent did not reduce the total consumption of isoflurane in patients undergoing sinonasal surgery [20]. We speculate that there are two reasons for these inconsistent results. First, isoflurane is a long-acting anesthetic gas when compared to sevoflurane [21]. Second, as the concentration increases, isoflurane results in a paradoxical increase in bispectral index (BIS) values [22].

Our study also showed that intraoperative MME consumption was significantly lower in the rocuronium/sugammadex group (0.067 vs. 0.076 mg/kg/h, *p* = 0.035). Opioids are the mainstay of perioperative pain management because of their analgesic effectiveness [23], and the calculation of MME allows the comparison of the potency of different types of opioids. One study showed that the use of the combination of rocuronium and sugammadex was associated with lower intraoperative remifentanil doses in patients receiving LMS when compared to the combination of succinylcholine and cisatracurium [24]. Another retrospective study conducted by Ezri et al. also showed that rocuronium/sugammadex use was associated with less intraoperative fentanyl consumption in patients undergoing laparoscopic sleeve gastrectomy [25]. However, other studies have reported contradictory results. Gu et al. conducted a randomized clinical trial that showed no significant difference (*p* = 0.538) in intraoperative remifentanil consumption between the rocuronium/sugammadex and rocuronium/neostigmine groups in laparoscopic colorectal surgery [26]. Therefore, we believe that further large-scale, high-quality trials are needed. Furthermore, during the postoperative period, the effect of rocuronium/sugammadex on opioid consumption has remained inconsistent. In our study, there was no difference in postoperative MME consumption between the two groups (*p* = 0.054). In contrast, sugammadex was associated with significantly greater postoperative MME consumption (*p* < 0.001) than neostigmine in patients undergoing laparoscopic gastric cancer surgery [27]. However, the result demonstrated by Gu et al. showed that the rocuronium/sugammadex group had less postoperative MME consumption (*p* < 0.001) than the rocuronium/neostigmine group in laparoscopic colorectal surgery [26]. Opioids have long been considered complementary to general anesthesia. Nevertheless, opioids are associated with well-known adverse effects such as ileus, delirium, sleep disturbance [28], respiratory depression, PONV [29], hyperalgesia [30] and the promotion of malignancies [31]. These recent issues have questioned the perioperative use of opioids, and intraoperative opioid use was therefore challenged by several clinical studies suggesting that opioid-free or opioid-sparing anesthesia may be more effective in providing adequate analgesia and reducing opioid-related adverse effects [29,32,33]. To our knowledge, to achieve the opioid-free or opioid-sparing anesthesia, opioids should be avoided during surgery and replaced by another hypnotics or analgesics to deal with surgical stimulations. Although the definition of opioid-free or opioid-sparing anesthesia differs in the literature and between institutions, nerve block, intravenous acetaminophen, ketamine, alpha-2 agonists, such as dexmedetomidine or clonidine, magnesium, and lidocaine alone or in combination have been proposed as an alternative to opioids [34,35].

Several complications have been reported with neostigmine, including recurarization, PONV, excessive salivation, and bradycardia [36]. At worst, cardiac arrhythmias and bronchospasm can even also happen [37]. Therefore, whether neostigmine should be routinely used to reverse neuromuscular blockade in each patient undergoing otorhinolaryngologic surgery is an important question for anesthesiologists. From this point of view, the use of sugammadex as a reversal agent seems to be a better choice.

The efficacy of sugammadex results from its ability to reverse neuromuscular blockade rapidly and completely, which has been shown to reduce postoperative pulmonary complications in several large-scale studies [9,38,39]. In addition to its benefit in enhancing recovery after surgery to the respiratory system, there has been much discussion on whether sugammadex has additional effects. PONV is associated with prolonged PACU stay [40], unplanned readmission [41] and patient dissatisfaction [42]. A randomized controlled trial conducted by Cappellini et al. demonstrated that the administration of sugammadex as a reversal agent was associated with a lower incidence of PONV than neostigmine in patients receiving LMS [12]. However, in an Egyptian study, the administration of sugammadex did not reduce the incidence of PONV in patients receiving septoplasty [43]. In our study, there was no significant difference in the incidence of PONV between the two groups. The current general consensus guidelines for PONV management conclude that the quality of evidence between sugammadex use and PONV incidence is limited due to unclear baseline risk among participants and involvement in open-label trials [44].

We also investigated the percentage of intraoperative hypertensive events (MAP > 120%, at 5-min intervals) and the intraoperative dose of antihypertensive agents between both groups. In our results, reversal with sugammadex did not increase the incidence of intraoperative hypertension compared with neostigmine (1.4 vs. 1.9 %, *p* = 0.325). However, the rocuronium/sugammadex group had significantly higher labetalol doses during surgery (*p* = 0.002). We conclude that the following reasons contributed to this result. Anesthesia time was longer in the rocuronium/sugammadex group (4.63 vs. 2.82 h, *p* < 0.001), resulting in a higher cumulative dose of rocuronium. Rocuronium has been widely reported for its vagolytic effect [45,46], resulting in an increased intraoperative heart rate, especially at high doses [47]. Therefore, this series of results made anesthesiologists to tend to use more labetalol.

There was no significant difference in intraoperative blood loss between the two groups in our study. This result is consistent with that of a previous study on patients undergoing functional endoscopic sinus surgery [20]. In addition, our study did not show a significant difference in the length of hospital stay between the two groups. In contrast, previous studies revealed that the administration of sugammadex reduced the length of hospital stay in major abdominal and thoracic surgeries [48,49]. We speculate that this may be because patients undergoing major surgery are at moderate to high risk of postoperative pulmonary complications [50,51], and sugammadex may reduce these risks [9].

### Limitations

The propensity score establishes a balanced dataset and allows for a simple and direct comparison of baseline covariates between the experimental and control groups. This study still had several limitations. The current study may have an inherent bias due to its retrospective nature. Due to the retrospective nature of the data, we could not predict whether the results would be confirmed in a prospective evaluation. In addition, only data from patients undergoing otorhinolaryngologic surgery at a single center were included. The number of participants was limited, and the results may be applied only to otorhinolaryngologic surgery. In Taiwan, the cost of sugammadex is not included in the National Health Insurance (NHI). Consequently, the total medication costs of anesthesia were significantly higher in the rocuronium/sugammadex group (209.3 vs. 56.3 USD/person, *p* < 0.001), compared with cisatracurium/neostigmine group (Supplementary Table S1). Our findings support that the combination of rocuronium and sugammadex can achieve the opioid-sparing anesthesia, thereby reducing adverse effects of opioids. However, we did not investigate the cost performance between both groups. Differences in clinical practice and healthcare systems should be considered to determine the general cost/benefit from the combination of rocuronium and sugammadex. Some promising monitoring for nociception, such as nociceptive level (NOL^®^) monitors (Medasense Biometrics Ltd., Ramat Gan, Israel) is still not available in our country [52]. In addition, due to the interference of the electromagnetic operating system on the BIS value [53], BIS monitors are not routinely in our otorhinolaryngologic surgery. Unfortunately, the cost of TOF (train-of-four) for neuromuscular monitoring is not also covered by National Health Insurance in Taiwan. Further prospective randomized controlled studies using NOL, BIS and TOF monitoring in each patient might offer a more precise guidance and evaluation of the consumption of intraoperative opioids and volatile anesthetics.

## 4. Materials and Methods

This retrospective observational study was conducted at Kaohsiung Chang Gung Memorial Hospital, a tertiary hospital in southern Taiwan. This study was approved by the Institutional Review Board (IRB) of the Kaohsiung Chang Gung Memorial Hospital (IRB approval number: 202101959B0). The requirement for informed consent was waived due to the retrospective nature of the study. The Strengthening the Reporting of Observational Studies in Epidemiology (STROBE) statement was applied, and this study complied with the applicable guidelines [54].

### 4.1. Data Collection and Study Design

A total of 2848 patients were intubated under general anesthesia for otorhinolaryngologic surgery at our center in southern Taiwan from January 2020 to December 2020. Medical records were retrieved from our database, and patients with the American Society of Anesthesiologists (ASA) physical status classification 5 (*n* = 2) and incomplete data (*n* = 198) were excluded. Two hundred patients were excluded. Therefore, 2648 cases were enrolled. One hundred and sixty-seven patients received rocuronium/sugammadex, and the other group received cisatracurium/neostigmine (*n* = 2481). Finally, 1:1 propensity score matching was performed for 238 patients, with 119 patients in each group. The propensity score matching was based on sex, age, body weight, and type of surgery (Figure 1).

### 4.2. Anesthesia Management

General anesthesia was induced in all patients who underwent otorhinolaryngologic surgery with intravenous fentanyl 2 mcg/kg and propofol 2 mg/kg mixed with lidocaine 20 mg. For the cisatracurium/neostigmine group, cisatracurium 0.2 mg/kg was administered during induction, while the rocuronium/sugammadex group received rocuronium 0.8 mg/kg during induction. All patients were intubated with an endotracheal tube, and sedation was maintained with sevoflurane intraoperatively. In our institution, the intraoperative hemodynamic status during surgery must be maintained within 20% of their normal range. Therefore, we defined MAP ≥ 120% as the intraoperative hypertension and initiated the interventions. Different types of opioids, including fentanyl, alfentanil, and morphine, were used for intraoperative analgesia during surgery, depending on the anesthesiologist’s preference. Antihypertensive agents, including labetalol and nicardipine, were administered according to the same principle. Finally, at the end of anesthesia, NMBA was antagonized with sugammadex (2 mg/kg or neostigmine 0.05 mg/kg) in the two groups. To minimize the cholinergic side effects of neostigmine, atropine 0.02 mg/kg was co-administered with neostigmine in the cisatracurium/neostigmine group. Glycopyrrolate was not available at our institution.

### 4.3. Primary and Secondary Outcomes

The primary outcome measure was sevoflurane consumption. On the other hand, the intraoperative MME consumption, intraoperative duration of hypertensive events, extubation time, incidence of delayed extubation, postoperative MME consumption, VAS score at the ward within 24 h, PONV in the ward within 24 h, and LOS were the secondary outcomes. The interval between the administration of the reversal agent and removal of the endotracheal tube was defined as the extubation time. Delayed extubation was defined as extubation performed outside the operating room, including the PACU or the intensive care unit. In addition, PONV prophylaxis was routinely performed based on patient risk factors, following the recommendations of the guidelines [44].

Sevoflurane consumption was retrieved from the electronic data recorded in the anesthesia machine, Primus (Drägerwerk AG, Lübeck, Germany), Carestation 620 (GE Datex-Ohmeda, Madison, WI, USA), Avance (GE Datex-Ohmeda, Madison, WI, USA), or S/5 ADU (GE Datex-Ohmeda, Madison, WI, USA). Opioids are widely used as supplements to general anesthetics and analgesic agents for intraoperative surgical stimulation. At our institution, several types of opioids, including fentanyl, alfentanil, and morphine, are administered intravenously during surgery. Therefore, we converted all opioid doses into MME, which is used to quantify the different types and routes of administration of opioids for a consistent comparison [55]. Pain assessment in the ward within 24 h was also evaluated. The VAS score (10 cm scale from 1 to 10; 0, no pain; 10, worst possible pain) is commonly used to assess postoperative pain. In addition, postoperative MME consumption and VAS scores in the ward were evaluated and recorded by certified registered nurse anesthetists within 24 h after surgery.

### 4.4. Statistical Analysis

Categorical variables, such as sex, ASA physical status, Apfel score, comorbidities, and type of surgery, are presented as raw numbers or percentages. The Chi-squared or Fisher’s exact test was used to compare the groups. Continuous numeric data were tested using the Student’s *t*-test (normality) or Mann–Whitney U test (non-normality) and are presented as the median (25–75%). The propensity scores were calculated based on sex, age, body weight, and surgery type. SPSS (version 22.0; IBM Corp., Armonk, NY, USA) was used for all statistical analyses. A *p*-value of <0.05 was considered to indicate statistical significance.

## 5. Conclusions

In patients undergoing otorhinolaryngologic surgery, the combination of rocuronium and sugammadex was found to be associated with significant preservation of intraoperative sevoflurane and MME consumption with a reduction of 14.2 and 11.8%, respectively, compared to cisatracurium/neostigmine. The use of the combination of rocuronium and sugammadex also significantly increased the dose of intraoperative labetalol (*p* = 0.002), although there was no significant difference in intraoperative hypertensive events between both groups. Our results may encourage the use of the combination of rocuronium and sugammadex as part of volatile-sparing and opioid-sparing anesthesia in otorhinolaryngologic surgery.

## Figures and Tables

**Table 1:** Demographic and clinical characteristics of patients in the cisatracurium/neostigmine and rocuronium/sugammadex groups.

Variables (Unit)	N(%)/Median (IQR)	Cisatracurium /Neostigmine *n* = 119	Rocuronium /Sugammadex *n* = 119	*p*-Value
Sex				
Female	67 (28.2%)	35 (29.4%)	32 (26.9%)	0.665
Male	171 (71.8%)	84 (70.6%)	87 (73.1%)	
Age	43.0 (31.0–56.0)	43.0 (29.0–57.0)	43.0 (33.0–54.0)	0.786
Body weight (kg)	75.0 (65.0–86.0)	75.0 (67.0–87.0)	75.0 (64.0–86.0)	0.649
ASA classification				
Ⅰ	20 (8.4%)	13 (10.9%)	7 (5.9%)	0.374
Ⅱ	181 (76.1%)	88 (73.9%)	93 (78.2%)	
Ⅲ	37 (15.5%)	18 (15.1%)	19 (16.0%)	
Apfel score				
0	27 (16.7%)	11 (13.9%)	16 (19.3%)	0.822
1	55 (34.0%)	27 (34.2%)	28 (33.7%)	
2	53 (32.7%)	28 (35.4%)	25 (30.1%)	
3	24 (14.8%)	11 (13.9%)	13 (15.7%)	
4	3 (1.9%)	2 (2.5%)	1 (1.2%)	
Hypertension				
No	195 (81.9%)	98 (82.4%)	97 (81.5%)	0.866
Yes	43 (18.1%)	21 (17.6%)	22 (18.5%)	
DM	(-)	(-)	(-)	
No	218 (90.3%)	106 (89.1%)	109 (91.6%)	0.510
Yes	23 (9.7%)	13 (10.9%)	10 (8.4%)	
CVA				
No	237 (99.6%)	118 (99.2%)	119 (100.0%)	1.000
Yes	1 (0.4%)	1 (0.8%)	0 (0.0%)	
Surgical indication for otorhinolaryngologic surgery				
Multiple sinusectomy	38 (16.0%)	17 (14.3%)	21 (17.6%)	0.830
Pansinusectomy	20 (8.4%)	12 (10.1%)	8 (6.7%)	
Septomeatal plasty	34 (14.3%)	18 (15.1%)	16 (13.4%)	
Uvulopalatopharyngoplasty	118 (49.6%)	59 (49.6%)	59 (49.6%)	
Oral tumor or oropharynx excision	28 (11.8%)	13 (10.9%)	15 (12.6%)	

Kolmogorov–Smirnov test (normal distribution), Mann–Whitney U test, chi-square test, Fisher exact test; IQR, interquartile range; ASA, American Society of Anesthesiologists physical status classification; DM, diabetes mellitus; CVA, cerebrovascular accident.

**Table 2:** Demographic and intraoperative and postoperative clinical presentation in patients in the cisatracurium/neostigmine and rocuronium/sugammadex groups.

Variables (Unit)	N(%)/Median (IQR)	Cisatracurium /Neostigmine *n* = 119	Rocuronium /Sugammadex *n* = 119	*p*-Value
Intraoperative				
Duration of anesthesia (h)	3.33 (2.35–6.17)	2.82 (2.08–4.33)	4.63 (2.92–6.75)	<0.001
Baseline MAP	97.2 (88.7–106.2)	98.7 (89.7–107.5)	95.8 (87.8–104.7)	0.354
Percentage of ^†^ MAP > 120% (%)	1.4 (0–6.4)	1.9 (0–6.5)	1.4 (0–5.9)	0.325
Fluid mL/kg/h	2.13 (1.63–2.67)	2.22 (1.66–2.89)	2.08 (1.50–2.56)	0.064
Sevoflurane consumption (mL/h)	0.20 (0.18–0.21)	0.21 (0.19–0.24)	0.18 (0.16–0.19)	0.009
^††^ Intraoperative MME (mg/kg/h)	0.073 (0.056–0.102)	0.076 (0.057–0.116)	0.067 (0.056–0.092)	0.035
Labetalol (mg)	0 (0–2.5)	0 (0–2.5)	0 (0–5.0)	0.002
Nicardipine (mg)	0 (0–1.0)	0 (0–0.5)	0 (0–1.0)	0.245
Postoperative				
Postoperative parecoxib (Dynastat^®^)	36 (15%)	21 (18%)	15 (13%)	0.278
^††^ Postoperative MME (mg/kg/h)	0 (0–0)	0 (0–0)	0 (0–0)	0.054
VAS at ward	2 (2–3)	2 (2–3)	2 (1–3)	0.086
PONV at ward	1 (0.4%)	1 (0.8%)	0 (0%)	1.0
Blood loss (mL)	0 (0–100)	0 (0–50)	0 (0–100)	0.507
Extubation time (min)	5 (5–10)	5 (5–10)	5 (5–10)	0.176
LOS (day)	4 (2–6)	4 (3–6)	4 (2–6)	0.553
Percentage of delayed extubation (%)	3 (1.3%)	3 (2.5%)	0 (0%)	0.111

Kolmogorov–Smirnov test (normal distribution), Mann–Whitney U test, Chi-squared test, Fisher exact test; IQR, interquartile range; MAP, mean arterial pressure; MME, morphine milligram equivalents; VAS, visual analog scale; PONV, postoperative nausea and vomiting; LOS, length of stay. ^†^ At 5-min intervals. ^††^ Opioid consumption was converted into MME.

## Data Availability

The data presented in this study are available from the corresponding author upon reasonable request.

## Supplementary Materials

The following supporting information can be downloaded at: https://www.mdpi.com/article/10.3390/ph15070894/s1, Table S1: Comparison of total medication costs of anesthesia.
