# Supplementary material for: Analgesic and Anesthetic Efficacy of Rocuronium/Sugammadex in Otorhinolaryngologic Surgery: A Propensity Score-Matched Analysis
Source: Pharmaceuticals (Basel). 2022 Jul 19;15(7):894. doi: 10.3390/ph15070894 (PMC9318942; doi:10.3390/ph15070894)
Supplement: Supplementary file 1 [file pharmaceuticals-15-00894-s001.zip › pharmaceuticals-1775741-supplementary.pdf]

Supplementary Table S1.

**Variables (unit)**	**N(%)/median (IQR)**	**Cisatracurium /neostigmine n = 119**	**Rocuronium /sugammadex n = 119**	***p*-value**
Medicine costs (USD/person)	162.5(157.6-209.7)	56.3(42.6-85.6)	209.3(180.7-231.2)	< 0.001

Mann-Whitney U test; IQR, interquartile range.
